# Assessing the interactions of people and policy-makers in social participation for health: an inventory of participatory governance measures from a rapid systematic literature review

**DOI:** 10.1186/s12939-023-01918-2

**Published:** 2023-11-17

**Authors:** Prateek Gupta, Benjamin Rouffy-Ly, Katja Rohrer-Herold, Kira Koch, Neethi Rao, Charlotte Poulussen, Lara Brearley, Hala Abou-Taleb, Dheepa Rajan

**Affiliations:** 1 Special Programme On Primary Health Care, World Health Organization, Av. Appia 20, 1211 Geneva, Switzerland; 2 Universal Health Coverage/Health Systems Department, World Health Organization, Magless El Shaab, PO Box No. 146, Cairo, 11516 Egypt; 3 European Observatory On Health Systems and Policies, Place Victor Horta/Victor Hortaplein, 40/10, 1060 Brussels, Brussels Belgium

**Keywords:** Participatory governance, Social participation, Measures, Metrics, Indicators, World Health Organization

## Abstract

Social participation, also termed stakeholder voice, is an important component of health system governance. Increased interactions between the community and policy makers could facilitate a more responsive health system that targets the needs of the community better. Recently, the World Health Organization (WHO) published a handbook on social participation that identified five key themes for ministries of health to consider when engaging the input of the community. In this rapid systematic literature review, we aimed to identify quantitative and qualitative measures that have been used to assess aspects of social participation involving people and policy makers. We identified 172 measures from 48 studies from countries in all six WHO regions. These measures were categorized by all five themes from the handbook on social participation and these measures are linked to 27 concepts. This rapid review found that the focus of measures is largely on the existence of participation—be it by the general population or specific vulnerable groups—rather than on the quality of their participation. The measures in this inventory may be useful for ministries of health and other key stakeholders to use when developing methods to assess and encourage social participation in their context.

**Supplementary Information:**

The online version contains supplementary material available at 10.1186/s12939-023-01918-2.

## Key messages

• The recent COVID-19 pandemic highlighted the critical importance of social participation in health system governance. Measuring social participation in health system governance would provide a measure of people’s input into health-related policies. Increasing the community’s participation could encourage improvements in the effectiveness and equity of health systems to achieve Universal Health Coverage by focusing on the needs of the community.

• Currently we have limited measures of social participation that could be used for decision making in health systems, limiting options that ministries of health have to assess social participation in their contexts and inform priorities for social participation in health systems.

• In this review, we describe 172 measures identified in the literature that have been used to collect empirical evidence on concepts related to social participation. In addition, we have categorized these measures according to a handbook on social participation published by the World Health Organization.

• This rapid review found that the focus of measures is largely on the existence of participation—be it by the general population or specific vulnerable groups—rather than on the quality of their participation.

• This inventory initiates the process to develop a monitoring framework for social participation that could be relevant to national or sub-national needs.

**Supplementary Information:**

The online version contains supplementary material available at 10.1186/s12939-023-01918-2.

## Introduction

### Inclusive decision-making as a core element of health system governance

The World Health Organization (WHO) has defined that governance [of health systems] “involves ensuring strategic policy frameworks exist and are combined with effective oversight, coalition-building, regulation, attention to system-design and accountability” [1]. It is generally understood, as highlighted in health system frameworks such as the Health System Building Blocks [2] or the Health System Functions [3], that health system governance is a cross cutting function that affects health system outcomes. Health system governance is broader than the government institutions responsible for its stewardship, [4] and it implies coordination and collaboration with actors and actions within and beyond the health sector [5].

A review of health systems governance frameworks highlighted that 14 frameworks out of 19 recognized some aspect of inclusiveness of the policy-making processes as a critical element of good health system governance [6]. Inclusive health system governance allows that all stakeholders have a chance to provide input on the direction of and decisions that affect the health system [5, 7]. Social participation focuses on participatory spaces designed to gather the community's and civil society’s input for decision-making. While actors such as health professional or private sector providers can also be involved in these spaces, engaging specifically with these groups comes with its own set of objectives, approaches and required capacities [8], which are beyond the scope of this review.

Social participation could facilitate societal consensus on relevant health topics, design strategies and policies that are responsive to population needs, support acceptance and ownership of decisions, improve implementation, and increase trust in government actors and public institutions [9]. Consequently, emphasizing social participation, as well as general stakeholder engagement, in health systems processes can shift governance from a top-down orientation to more responsive policy, planning and implementation processes [10], fostering more equitable and effective health systems [5]. In line with the Health System Performance Assessment (HSPA) framework and to support countries in achieving inclusive governance goals, WHO has developed its handbook on social participation for UHC to provide countries with guidance for creating and sustaining spaces for meaningful participation [9]. This handbook recommends that ministries of health enable social participation through actions in the following five themes:

- Selecting participants which are considered legitimate and able to **represent** a constituency, themselves/their own experience, or an idea;
- Ensuring that all stakeholders (those organizing as well as those participating) have the necessary **capacities** to engage meaningfully with each other;
- Considering the options and prerequisites for **policy-uptake** of recommendations stemming from participatory mechanisms;
- Accounting for the relevance of (or the absence of) **legal frameworks** for social participation; and
- Considering the prerequisites (participatory space features and sub-functions) which contribute to **participatory engagement being sustained over time** [9].

These themes, individually and in combination, provide options to diminish underlying societal power imbalances hindering meaningful social participation in health system decision-making. Given their centrality for successful government engagement with civil society, communities, and the population, these five themes were used to a) orient the search strategy and define key search terms and b) to structure the results section of this systematic literature review.

### Measuring the meaningfulness of social participation: a stepping stone to move from commitment to implementation

The involvement of communities in their health is integral as part of a rights-based approach [11], and, over the years, countries have repeatedly committed to the principle of social participation. Such commitments include a number of high level documents from the Sustainable Development Goals (SDGs) agenda—with targets for governments to ensure “responsive, inclusive, participatory and representative decision-making at all levels” as in SDG 16 [12] to the more recent political declaration on Universal Health Coverage where states have committed to “engage all relevant stakeholders, including civil society, private sector and academia, as appropriate, through the establishment of participatory and transparent multi-stakeholder platforms and partnerships, to provide input to the development, implementation and evaluation of health- and social-related policies and reviewing progress for the achievement of national objectives for universal health coverage, while giving due regard to addressing and managing conflicts of interest and undue influence” [13].

While almost all countries have committed to the principle of social participation, details on implementation of social participation in health is lacking and developing more standardized measures for social participation will help monitor how countries are faring in their commitments. In addition, a set of measures will be helpful for inclusion into a potential World Health Assembly resolution on social participation which several WHO Member States are currently preparing. The resolution can be more useful if a monitoring and evaluation framework is embedded within it to ensure that countries go beyond obligation to the principle of participation to concrete action towards fulfillment of a more participatory modus operandi of the health sector. Monitoring and evaluating social participation in health governance is crucial to assess the current status, identify gaps and opportunities to focus investments, promote transparency, hold government accountable for commitments made, and sustain political interest.

However there is no common approach or terminology in regard to social participation for decision-making [9] and only limited empirical evidence documented to date for measurement of social participation in the health sector [10]. In this review, social participation is meant to be an encompassing term to denote individuals, populations, communities, and civil society acting in some manner for health system governance [9]. In addition, social participation may not be limited to a discrete time period or component of the policy making and policy implementation continuum. Sustainable and responsive policy-making is a dynamic process, all of which makes measuring the inclusion of civil society and community voices a challenge [10]. This is further complicated by the interplay of various societal, political and power-related factors involved [5].

The WHO HSPA approach provides topical areas which should be assessed, but not specific measures relevant for linking the stakeholder voice sub-function of governance to the achievement of final health system goals such as equity, efficiency, and financial protection [5]. Building on this approach, and on the five themes put forward by the WHO handbook on social participation for UHC [9], this review is intended as a first step towards the development of a standardized set of measures to provide countries with the means to monitor their progress in institutionalizing social participation.

### Aims and objectives: closing a knowledge gap

This paper intends to address a knowledge gap by analyzing how social participation in health has been monitored and measured since 2000, and propose some next steps towards a comprehensive social participation monitoring and evaluation framework.

The objectives of this paper are to:

1. Perform a rapid systematic review of the literature on measures used to assess social participation;
2. Map existing measures to the main themes to be reflected on to ensure meaningful participation put forward in the WHO handbook on social participation [9];
3. Build a repository of measures providing all stakeholders with options to measure the different themes of social participation in health; and
4. Propose some next steps to develop a validated set of measures for the stakeholder voice sub-function of the governance function to inform future health system assessments.

To ensure conceptual consistency for this rapid systematic literature review, we rely on terminology on social participation and power as put forward by WHO [9] and on terminology on broader aspects of governance as defined in WHO´s HSPA framework [5].

## Methods

We applied a rapid review approach, following standardized methods and reported in accordance with PRISMA guidelines [14–17].

### Information sources and search strategy

Previous works on health systems building blocks, social participation, health system functions, and health systems assessments informed the development of the search strategy [5, 9, 18, 19]. While social participation has been described in the public health literature, the terminology used to describe concepts of social participation varies. The term ‘social participation’ itself was chosen in the WHO-published document as it was the most expansive of options. That handbook, used as a framework for analysis for this review, noted a diversity in use of participation-related terminology in the literature, even when describing similar themes and sub-themes. For example, the term community can refer to a group of individuals who share common socio-demographic features, individuals who associate in support of a common cause, or individuals who share an interest [9]. The search terms used in this review attempted to capture that diversity such as the use of the terms ‘representativeness’, ‘hard-to-reach groups’, among other terms.

In addition, the authors contributed key terms to include in the database search strategies. To identify articles published since WHO’s conceptualization of the health system functions in 2000, six databases were searched on 10 February 2022 initially: CINAHL, Embase (using OVID); MEDLINE (using OVID); Global Health (using OVID), Scopus, and Web of Science; covering the period from 01/01/2000 to 12/31/2021. We devised the search syntax by identifying key words related to three concepts: 1) social participation, AND 2) health system assessments, AND 3) health systems governance. Searches were not restricted by geographical region or income status and all search terms were in English. The complete list of search terms used for all the databases are presented in Additional file 1.

Additional studies were identified through citation searches of seven health system assessment tools [19–25] and used the ‘cited by’ function in Google Scholar to identify subsequent studies that had cited the reviews. We conducted forward and backward screening of all articles in the full-text screening phase and relevant publications (e.g., guidelines, tools, reviews, opinion pieces) to find any additional studies fitting the inclusion criteria. If any literature reviews were identified in our search, we screened the citation lists of those reviews to identify articles that fit our criteria. Finally, to ensure no key publications were missing, the study authors and representatives from the authors institute were consulted to identify any additional studies that may have been missed.

### Eligibility criteria

Studies conducted in any country that included and defined a measure of social participation related to health system governance were eligible for inclusion. In each study, items considered as pre-requisites for social participation were also identified and categorized. No restrictions were placed on measures used by the authors of the study.

### Selection process

Retrieved title and abstract records were loaded into the reference manager programme Zotero and duplicate references were removed [26]. The two first authors initially double screened 5% of the records to ensure consistency in selection between the reviewers. The two first authors then independently screened the remaining titles and abstracts and cross-checked with each other in case either author was uncertain of a specific document’s inclusion.

Full-texts of potentially relevant studies identified from the title and abstract screening were obtained and screened by both the two first authors, with any uncertainties discussed and resolved between the two first authors. Where we were unable to access the full text, we attempted to contact the study authors via email. The reason for excluding studies based on full-text review was recorded.

### Data collection process and risk of bias assessment

Study information was extracted into a pretested Microsoft Excel-based extraction tool to capture data on how the measures were constructed and defined, which individual items were included, the methods for construction of any composite scores and the data sources used.

Given the review’s focus, data on the study results was not extracted and a formal quality assessment or risk of bias assessment of each study was not undertaken. Information from included studies was extracted by the two first authors and discrepancies were identified and resolved by discussion between them.

### Categorization of measures

We updated the terminology used in the WHO handbook on social participation to categorize the measures identified in this study as follows in the parentheses: 1) representation in participation (representativeness); 2) capacities for meaningful government engagement with the population, communities and civil society (capacities); 3) from population engagement to decision-making (policy uptake); 4) legal frameworks for participation and 5) sustaining participatory engagement over time (sustainability). For the capacities theme, we used terminology from the handbook to develop sub-themes. As we reviewed the measures, we inductively developed concepts for an additional level of categorizations and linked these concepts to the themes and sub-themes [9].

## Results

### Summary of search

**Fig. 1:**
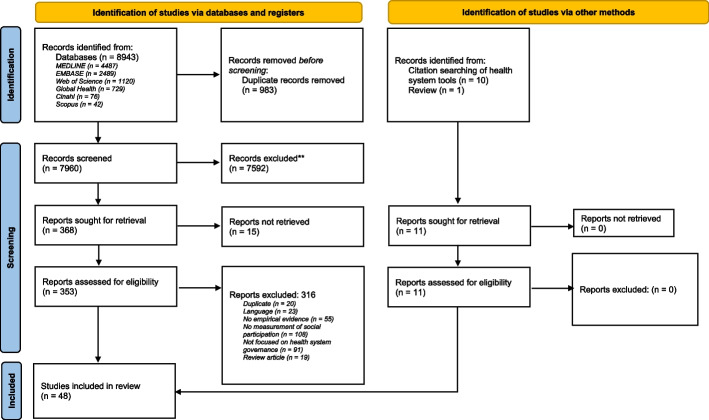
PRISMA Flow Chart

Through the database search, we identified 8,943 records. After removing duplicates, we screened 7,960 documents, of which 368 were considered for full-text review. As noted in the PRISMA Flow Chart (Fig. 1), we were not able to retrieve 15 articles despite contacting the authors directly for a copy. Of those papers assessed in the full-text review, 37 studies were identified as eligible for inclusion. The 316 reports excluded were due to being duplicates (*n* = 20), in a language other than English or French (*n* = 23), lacking any empirical evidence (*n* = 55), not including any measurement of social participation (*n* = 108), not focused on health system governance (*n* = 91), or being a review article (*n* = 19). We screened the reference lists of the review articles to identify additional articles and one article met our criteria. In addition, through the citation search process of the seven health system assessment tools, we identified 10 reports to include in this literature review.

Since 2000, 48 reports met the inclusion criteria of this paper and at least one paper was published every year except for the years 2004 and 2021. When categorizing the studies by WHO region, the Region of the Americas had the most studies with 17, followed by African Region (*n* = 12), the European Region (*n* = 8), the South-East Asia Region (*n* = 6), the Western Pacific Region (*n* = 3) and the Eastern Mediterranean Region (*n* = 1). Studies from the United States of America and Canada were the largest number of studies from a single country (*n* = 5), and other countries with multiple studies included Mexico (*n* = 3), Nepal (*n* = 3) Ghana (*n* = 3), Tanzania (*n* = 3), Sweden (*n* = 2), and Zambia (*n* = 2). Two studies were conducted in multiple countries, one study was global in nature, and one study focused on countries in Europe. When categorizing the studies by income status, 20 studies from high-income countries were published, followed by lower-middle income countries (*n* = 16), upper-middle income countries (*n* = 8), and finally low-income countries (*n* = 3). A majority of the studies had a subnational focus, with seven at the provincial/state level (*n* = 12), province/district (*n* = 1), district only (*n* = 11), sub-district that also includes villages and communities (*n* = 8), and municipalities (*n* = 9). Five studies focused on national level participatory governance and two studies were on a mix of national and district level processes. Additional detail is provided in the Supplementary Table 1.

### Inventory of measures

Based on the extraction of key details of the different measures used in the 48 studies, we created a detailed table (see supplementary table 2) clustering similar measures and highlighting data type and the qualitative/quantitative nature of the measure. The clusters were designed by empirically grouping existing measures measuring the same concept. Measures could be counted twice if the same question could assess two different social participation themes or sub-themes depending on the data source (e.g. depending on who the survey respondent is, a self-evaluation of the “ability to compromise” could be considered a measure of government or population communication skill). In total, 172 measures were identified, inventoried, clustered and mapped as summarized in Table 1 against the themes that render participation meaningful as identified in the WHO handbook on social participation [9].

**Table 1:** Summary table of measures with number of measures categorized by concept and counted by qualitative or qualitative data collection method

Social participation theme	Sub theme	Concept	Qualitative measures (n)	Quantitative measures (n)	Total measures (n)
*Capacities*	Government communication skills	Ability to listen	1	1	2
*Capacities*	Government communication skills	Ability to negotiate with civil society	1	1	2
*Capacities*	Government communication skills	Ability to provide feedback	1	0	1
*Capacities*	Government communication skills	Clarity of communication	3	2	5
*Capacities*	Government communication skills	Public awareness of the participatory space	0	7	7
*Capacities*	Government recognition skills	Perceived added value of participation	4	3	7
*Capacities*	Government technical skills	Participant perception of facilitation	5	3	8
*Capacities*	Government technical skills	Participant perception of space design	14	3	17
*Capacities*	Population communication skills	Ability to negotiate	0	1	1
*Capacities*	Population communication skills	Ability to speak publicly	1	1	2
*Capacities*	Population recognition skills	Perceived usefulness of participation	5	6	11
*Capacities*	Population recognition skills	Perception of empowerment	3	2	5
*Capacities*	Population technical skills	Capacity to engage	6	3	9
*Capacities*	Population technical skills	Technical knowledge of the issue	1	1	2
*Legal framework*	N/A	Documented procedures and strategies for participation	2	1	3
*Legal framework*	N/A	Participatory spaces delineated in laws and programs	2	2	4
*Policy uptake*	N/A	Documented impact on decision-making	0	3	3
*Policy uptake*	N/A	Link to downstream changes	1	3	4
*Policy uptake*	N/A	Perceived impact on decision-making	16	9	25
*Representativeness*	N/A	Diversity	6	9	15
*Representativeness*	N/A	Participant perception of clarity of roles	3	1	4
*Representativeness*	N/A	Participant perception of the quality of representation	10	4	14
*Representativeness*	N/A	Proportion of different stakeholder groups	0	7	7
*Sustainability*	N/A	History of participation	1	2	3
*Sustainability*	N/A	Political will	1	0	1
*Sustainability*	N/A	Resources for participation	3	3	6
*Sustainability*	N/A	Sustained attendance	0	4	4
**Total**			90	82	172

### Representativeness

We identified 40 measures looking at the representativeness theme of participatory spaces. Examining which stakeholder groups are represented, how representants have been selected and whether they account for the diversity of the constituency that is being consulted is at the heart of the issue of credibility and perceived legitimacy of a participatory process [9].

Fifteen measures assessed the diversity of stakeholders in the participatory space through the presence of specific stakeholder groups [27–34], participant characteristics [35, 36] or the mere presence of general public [37, 38]. These measures were collected either quantitatively from cross-sectional surveys and document reviews (e.g. meeting minutes, reports) or qualitatively using in depth interviews, survey, document reviews or a mix of the three. An additional seven quantitative measures, mostly based on document reviews, measured the proportion of specific stakeholder groups represented in a participatory space, groups such as women [39], different castes [39], service providers, community members, church representatives, school staff, and more [40, 41].

The quality of the representation was addressed through four measures investigating the perceived clarity of roles and responsibilities among the different stakeholders [27, 29, 42, 43] and another 14 investigating all participants’ perception of representativeness. Perception of representativeness covers whether participants, both from the population and government, felt that the selection of representants was appropriate [44] and brought together relevant [33, 45] and legitimate [46] stakeholders representing the full variety of interests in the community [47]; or that specific stakeholder groups like women [42] or community and NGO leaders [48], different sectors [43] and civil society at large [49–53] were effectively involved. These measures related to perceptions of participants either used Likert scale surveys to quantify perception, or in-depth interviews focusing on a qualitative assessment.

### Capacities

With 79 measures, the necessary capacities for government officials and population to meaningfully engage with each other provides the largest number of measures in any of the five categories. Meaningful participation requires that all stakeholders involved are able to adequately fulfil their role. It requires that they all understand the importance and potential beneficial aspects of participation, the policy question being addressed and how it affects them and the people they represent, and that they all are able to influence the dialogue on an equal footing. For the purposes of this analysis, the capacity theme is divided into the sub themes of recognition, technical and communication skills, both for the population and government officials due to the large number of measures identified and because the sub-themes were already described in the handbook [9].

Eleven measures were linked to the population’s recognition skills focused on the perceived usefulness of participation by the community [35, 43, 45, 46, 50, 53–57]; and the population’s perception of empowerment [five measures] [51, 58], including three looking specifically at issues of trust [46], self-confidence [59] or ownership of the participatory space [60].

We found that population technical skills were investigated through nine measures around the capacity to engage [31, 33, 49, 61], with four specifically looking at prior training or experience engaging with social participation spaces [31, 36, 62]; and two measures investigating the participants technical knowledge of the issue discussed [36, 45].

Focusing on population communication skills, one quantitative measure (self-evaluation survey) looked at the participant’s ability to negotiate [46], while two assessed the ability to speak publicly using discourse analysis of meeting minutes [59] or the percentage of meetings where members of a specific minority group raised issues according to a cross sectional survey [29].

Government recognition skills measures focus mostly on the perceived added value of participation (seven measures) whether in principle [35, 53, 63], in the government self-interest [46], or as a key determinant of quality in policymaking [57].

Government technical skills are assessed, both qualitatively and quantitatively, through the perception of the participants regarding two main aspects: facilitation and space design. The eight measures looking at the facilitation skills focus on how the different stakeholder views are captured [64], how the public’s questions are answered [41], how the opportunities to speak up were distributed among participants [29, 38, 45], and other mechanisms used to enhance meaningful dialogue [42, 56, 65]. The 17 measures assessing space design focus on mechanisms to mitigate barriers to participation [31, 33, 43, 50, 62, 66] and to foster extensive participation [29, 35, 42, 44, 64, 67, 68].

Finally, government communication skills prior to the participatory activity were assessed by the public’s awareness of the existence of the participatory space (seven measures) through quantitative surveys using closed-ended questions and Likert scales [38, 50, 62]. Another five measures assessed the clarity of communication through communication material [36, 42, 44, 45] and regarding the stated objective of the space [36]. Communication during and after the participatory activity was also assessed through two measures on the ability to listen [46, 59], two measures on the ability to negotiate through compromise [46] and conflict-resolution [49], and one measure on the ability to provide feedback [41].

### Policy uptake

Ideally, data should inform dialogue, and decision-making should take into account both data and dialogue [69]. The 32 policy uptake measures referenced here aim to measure the perceived and documented links between dialogue and decision-making, and their direct and distal effects. Therefore, and while it is not the only objective of participation for health, policy uptake is a key outcome of the participatory mechanisms that we set out to explore.

We identified 25 measures looking at the perceived impact on decision-making, assessing either the participant’s perceived influence [50–52, 56, 58, 67, 68, 70, 71], or the influence of the community at large [28, 37, 43, 47, 48, 50, 61]. Like most measures assessing participant’s perception in this review, quantitative measures (nine) use Likert scale surveys, while 16 qualitative measures use in-depth interviews and focus groups.

Three additional quantitative measures examined the documented impact on decision-making through proportion of decisions made and programmes implemented that reflect the recommendations from a participatory space [38, 54], and the type of evidence used in decision-making according to surveyed decision-makers [30].

Another four measures look at more downstream effects of the participatory space, highlighting the percentage of increase in activities, targets and budget allocations for community identified priorities [62, 72, 73], or reviewing meeting minutes and health reports to qualitatively retrace the chronological link between participation and change [59].

### Legal framework for participation

Participatory spaces can emerge with or without being institutionalized and building on a legal framework is not a guaranty of a quality participatory space. However the presence and more importantly the type of legal framework, if in adequation with the country context, can reflect a participatory culture and be a potent tool for civil society to claim their right to health and participation.

In this review only seven measures out of 172 refer to the legal framework for participation making it the least investigated social participation theme. Four measures examine if and how participatory spaces were formalized, whether through committing agreements [74], being embedded in program and community planning [68, 75], or more generic normative and legal documents [67]. These four measures are not based on the review of legal documents but on surveys and interviews with government officials, the latter using a 1 to 4 rating scale to quantify the strength of the legal framework.

An additional three measures look at the existence of documented procedures and strategies to conduct participation [38] and how they promote or facilitate it in practice [28, 43]. The former is a qualitative assessment based on a document review of different plans and reports, the latter is a quantitative assessment on a 1 to 10 scale based on observations of the participatory activity.

### Sustainability of participatory engagement

“Sustaining participatory engagement over time implies ensuring long-term motivation, interest, capacity, and funding for participatory spaces by all stakeholders” [9]. It is instrumental in building a trusting relationship necessary for meaningful engagement.

Long term motivation and interest are reflected through four quantitative measures assessing the sustained attendance of participants according to documents such as meeting minutes [39, 51, 54] and household surveys around open-to-all municipal meetings [73].

Six measures assessed necessary resources for sustained participation such as capacity building and technical support [28, 31, 33], funds, material and time [46, 59], using a mix of survey, interviews and review of documents such as budgets of local authorities.

Finally, the history of participation in the community was examined quantitatively through two survey-based measures [46]—with one focusing on whether meetings are being held as planned [29]—or qualitatively through interviews discussing the continuity of community participation [65]. One additional measure examines the political will, asking during interviews about the local leadership support for participation [60].

## Discussion

### Social participation is being measured, predominantly at the level of its existence rather than of its quality but there is no consensus

The first important finding is that social participation in health is being measured and has been since at least the year 2000. This review focused on measures used empirically to assess social participation in health since the year 2000, looking specifically at the interaction between the “people” and “policymakers” spheres. However, no unified approach has emerged amongst academics and practitioners as to how best to measure social participation. This lack of consensus is illustrated by the fact that no tool in this review has been used in more than one study, and that most of the tools and measures inventoried here are of the authors’ own design for the purposes of conducting the study.

While there is no global standard on how to measure social participation given the heterogeneity of measurement options identified in this review, it is interesting to note that the different tools that the various authors have built for their studies are measuring common concepts. In fact, from the 172 measures extracted from our review, we were able to cluster them down to 27 measurement groups, all of them fitting under the five themes of social participation. This demonstrates that while the work of unifying the different approaches to measuring social participation in health is yet to be achieved, the various stakeholders in the field have a similar understanding of the key dimensions to assess. It is also worth noting that these measures are not evenly spread amongst the different themes. Indeed, 151 out of 172 measures are focused on who is engaging and how (i.e. representativeness and capacities, 119 measures), and to what effect (i.e. policy uptake, 32 measures). This last part can be explained to some extent by our selection bias towards studies looking at participatory spaces for which the goal was decision-making. On the other hand, longer term aspects and underlying determinants of the participatory space (i.e. the legal frameworks and sustainability dimensions, 21 measures) seem to be less of a priority for researchers.

Another key point here is that the focus of these measures is largely on the *existence* of participation—be it by the general population or specific vulnerable groups—rather than on the quality of their participation. For example, the presence of different participants in a participatory space is picked up by these measures, but *how* the participants interacted and were heard less so. For the meaningful aspect of participation to be measured, it must be (better) defined. Data might need to be collected through both objective measures (speaking time of different participants, for example, or a dissection of whose views exactly influenced decisions) as well as subjective ones (participant views on whether they felt listened to), the latter being somewhat more complicated to do and standardize.

### A diverse measurement landscape for social participation exists, but without standardization

We found that data on social participation was quite rich and diverse. We were able to build a repository of measures that identifies the data collection method for each measure, and sources for these measures include group discussion, key informant interviews, surveys and reviews of various types of documents (e.g. meeting minutes, participant list, laws and other regulatory texts). While each study explicitly mentioned their data source and collection method, the exact nature of the measure was not always clear (e.g. exact wording of a question, components of a composite measures), and tools not always provided in annex to the publication. The measures we extracted are quite diverse in data source and measurement options. The ratio of qualitative (90) versus quantitative (82) measures is surprisingly balanced considering several aspects of social participation are qualitative in nature. This is illustrated by measures focusing on the perception of the different stakeholders regarding different aspects of a specific participatory process such as perception of representation [42, 49] or perceived usefulness of participation [35, 46, 53]. These measures compose the majority of identified measures (95/172), they are by their very definition quite subjective, and they are of critical importance in assessing social participation seeing as participants’ perception of a process is intimately linked to the trust they have in said process which in turn impacts directly how forthcoming they are during a consultation and how likely it is that they will demonstrate sustained interest over time [9]. This high number of quantitative measures can be largely explained by the fact that numerous authors resorted to using Likert scales to assign numeric value to these qualitative concepts, allowing them to treat such measures as quantitative and conduct statistical analysis of the data collected. In contrast, some measures were more clearly quantitative in nature looking at issues like proportion of participants from specific stakeholder groups [39], or proportion of programmes endorsed by a participatory space being implemented [54].

In summary, we categorized the 172 measures into the five social participation themes as laid out in the WHO Handbook on social participation [9]—however, the formulation and emphasis given for each particular question or measure were not common across them all, which means that comparing data across different measures within the same category would not really be possible, or at the very least, fraught with many caveats. Future work in this area should aim to identify a core set of measures that are linked to a key characteristic of a theme.

To build on this repository, it would be beneficial to screen the grey literature for various assessment tools used by local and national authorities and their technical partners to assess social participation in health systems governance. In addition, validation exercises for a sub-set of the measures at country level could help the process of prioritization of the minimum set of measures per social participation theme.

### Policy use of social participation data seems to be fairly low

WHO’s handbook on social participation notes that improved decision-making regarding participatory governance can occur when there is a dialogue based on data [9]. This review has identified limited evidence of the use of the data collected from the measurements of social participation by ministries of health to improve governance of health systems. Sixteen of the studies identified in this review include a member of government as an author [28–30, 32, 33, 39, 45, 46, 48, 56, 57, 60, 63, 67, 68, 71], but only two of the studies [45, 71] noted within the study that the government reviewed or used any of the collected data. Only six studies provide more than a one-time measurement providing an opportunity to assess changes over time [29, 55, 56, 63, 71, 73]. This widespread approach of looking at a specific space at a specific time could also explain why legal frameworks and sustained engagement are dimensions that have been generally overlooked.

In general, there is limited evidence of policy uptake as a result of a participatory process [9]. This points to a low political prioritization accorded to participation in health, which sheds light on a possible reason for low usage of participation-related monitoring data. This reinforces the need for more global health advocacy for concrete action on participation, such as the on-going efforts to work towards a World Health Assembly resolution on social participation. In addition, more research is needed to identify what the ministries of health consider as being important for their decision-making process and how well these or other measures can be used to collect policy-relevant data for decision-making.

### Social participation is largely measured at sub-national and local levels, through a single participatory process

In the HSPA for UHC Framework, social participation, labelled as stakeholder voice, is one of four subfunctions of governance that includes policy and vision, information and intelligence, and legislation and regulation [76]. The ultimate goal of our research agenda is to provide a set of measures that can assess the stakeholder voice sub-function of governance, however, all the studies included in this review put forward a set of measures for the different dimensions of specific participatory spaces, but none of them address the health system as a whole. In addition, given that most of the studies in this review were subnational in focus (32/48), it is unclear how to make inferences for the state of participatory governance in a country from data collected from a single, sub-national participatory space. An important part of the research agenda to build a set of measures for social participation in the health system will need to balance the need to account for participatory processes both at the national and subnational level, the constraints of what data can be effectively and reliably collected, and to elucidate how inclusive participatory governance mechanisms influence health systems goals such as equity and people-centeredness.

## Limitations

This work is intended to lay a foundation on which to build a set of measures for the stakeholder voice sub-function of health systems governance. While we stand by the boundaries we set for our search to match that research question, we acknowledge that these led us to ignore some potentially relevant work.

We did not engage with the rich existing literature in political science as a whole with foundational progress such as the work of Arnstein conceptualizing the ladder of citizen participation as early as 1969 [77]. Similarly we acknowledge that we did not identify measures of citizen participation in other sectors where citizen participation is well established, such as education [78] and the environment [79].

By setting the starting point of our review in 2000, we excluded early work on social participation in health. However, if such work was being used or adapted since then, it featured in our review. For example, Rifkin’s spidergram was not originally included as it was published prior to 2000 [20], but we included documents that cited it via Google Scholar [27, 31, 32, 43, 44, 65]. By searching only English and French documents potentially excluded a wealth of evidence on this agenda globally.

We excluded papers that focused on participation spaces in the provider-population interface, as they are widely explored as a mean to enhance quality of services and did not match our research agenda. Because we ensured inclusion of formal policymakers, we may have missed documents that focused on participatory spaces in fragile and conflict settings.

While we only included measures for which there was evidence of use, thus excluding theoretical measures that are yet to be field tested, we have not conducted a quality evaluation of the measures presented in this inventory and therefore do not specifically recommend one over the other.

Finally, we acknowledge that by focusing on the articles published on peer-reviewed journals, we did not review the wealth of assessment tools developed by specific stakeholders such as national and local authorities, NGOs, international agencies or bilateral donors. Reviewing such tools in the future will be a next step for this research agenda and will enrich this inventory. In addition, information on how social participation evolves over time in each country context may not have been documented as well in the documents that we reviewed.

## Conclusion

This literature review identified 172 measures of social participation in health while also highlighting the gap that exists in terms of having a normative standard for measuring social participation. This review framed the measures against five themes to provide options for program managers to consider when assessing social participation in their context with further categorization against sub-themes that were either deductively identified in the WHO handbook or inductively determined when categorizing the measures. While the wordings of the measures described in this review are specific to their context, the data collection methodologies that these measures use and concept aimed to be captured by the measure may be adapted to measure other themes and sub-themes in different contexts. This work is a first step towards development of a monitoring and evaluation framework for social participation in the health which can feed into an assessment of the governance function as part of a health system performance assessment.

The next steps for this area of work should be to widen the search to assessment tools for governance and other related topics used at the country level by the different stakeholders, especially ministries of health and their technical partners, to enrich the repository of measures. Once the repository is more complete, quality assessment exercises through expert consultations and field testing in countries will be needed to narrow down the set of measures to a number which is realistic in terms of data collection at country levels, and usefulness for decision makers to monitor participatory governance. This area of assessment is not likely to yield a narrow set of measures that can be applied universally. Instead, it may be useful for program managers in different countries and regions to identify which measures are linked to their local priorities. The choice to use specific measures from this inventory in a different context would benefit from a clear statement of the program managers’ information needs and the desired change.

### Supplementary Information

**Additional file 1.**

**Additional file 2.**

**Additional file 3.**

**Additional file 4.**

## Data Availability

No datasets were generated or analysed during the current study. The PRISMA checklist used for this study is included in Additional file 4.

## Abbreviations

WHO: World Health Organization
HSPA: Health Systems Performance Assessment
