# Supplementary material for: Assessing the interactions of people and policy-makers in social participation for health: an inventory of participatory governance measures from a rapid systematic literature review
Source: Int J Equity Health. 2023 Nov 17;22:240. doi: 10.1186/s12939-023-01918-2 (PMC10657134; doi:10.1186/s12939-023-01918-2)
Supplement: Supplementary file 2 — Additional file 2. [file 12939_2023_1918_MOESM2_ESM.docx]

Table 2 Location of studies included in review disaggregated by country

| Country | Studies |
| --- | --- |
| Afghanistan | (Anwari *et al.*, 2015) |
| Australia | (Brandstetter *et al.*, 2014) |
| Benin and Senegal | (Paul *et al.*, 2020) |
| Canada | (Boyce, 2001; Abelson *et al.*, 2007; Restall *et al.*, 2011; Regier *et al.*, 2014; Damani *et al.*, 2016) |
| Colombia | (Mosquera *et al.*, 2001) |
| Cuba | (Yassi *et al.*, 2003) |
| Denmark | (Scheele *et al.*, 2019) |
| Ecuador | (Buele *et al.*, 2020) |
| EU | (Fischer *et al.*, 2013) |
| Finland | (Simonsen-Rehn *et al.*, 2006) |
| Germany | (von dem Knesebeck *et al.*, 2002) |
| Ghana | (Owusu, 2011; Atinga *et al.*, 2019; Owusu *et al.*, 2019) |
| Global | (Peersman *et al.*, 2009) |
| Guatamala | (Ruano, 2013) |
| India | (Rao *et al.*, 2017) |
| Kenya | (O’Meara *et al.*, 2011) |
| Malawi | (Masefield *et al.*, 2020) |
| Mexico | (Arredondo and Orozco, 2006, 2008; Arredondo *et al.*, 2015) |
| Nepal | (Bishai *et al.*, 2002; Gurung and Tuladhar, 2013; Gurung *et al.*, 2018) |
| New Zealand | (Eyre and Gauld, 2003) |
| Spain | (Barbieri *et al.*, 2018) |
| Sweden | (Andersson *et al.*, 2005; Rosen, 2006) |
| Tanzania | (Shayo *et al.*, 2012; Pancras, 2016; Madon *et al.*, 2018) |
| Thailand | (Rajan *et al.*, 2019) |
| The Philippines | (Ramiro *et al.*, 2001) |
| Timor Leste | (Soares, 2013) |
| Uganda | (Kapiriri, 2017) |
| United Kingdom | (South *et al.*, 2005) |
| United States of America | (Wallerstein, 2000; Peterson *et al.*, 2007; Garza *et al.*, 2009; Weiss *et al.*, 2010; Truiett-Theodorson *et al.*, 2015) |
| Zambia | (Vian *et al.*, 2020, 2022) |

Supplementary Tables

Supplementary table 1 Included studies disaggregated by country income status as designated by the World Bank

| Country Income status | Study |
| --- | --- |
| *Lower Income* | (Anwari *et al.*, 2015; Kapiriri, 2017; Masefield *et al.*, 2020) |
| *Lower middle income* | (Ramiro *et al.*, 2001; Bishai *et al.*, 2002; O’Meara *et al.*, 2011; Owusu, 2011; Shayo *et al.*, 2012; Gurung and Tuladhar, 2013; Soares, 2013; Pancras, 2016; Rao *et al.*, 2017; Gurung *et al.*, 2018; Madon *et al.*, 2018; Atinga *et al.*, 2019; Owusu *et al.*, 2019; Paul *et al.*, 2020; Vian *et al.*, 2020, 2022) |
| *Upper middle income* | (Mosquera *et al.*, 2001; Yassi *et al.*, 2003; Arredondo and Orozco, 2006, 2008; Ruano, 2013; Arredondo *et al.*, 2015; Rajan *et al.*, 2019; Buele *et al.*, 2020) |
| *High Income* | (Wallerstein, 2000; Boyce, 2001; von dem Knesebeck *et al.*, 2002; Eyre and Gauld, 2003; Andersson *et al.*, 2005; South *et al.*, 2005; Rosen, 2006; Simonsen-Rehn *et al.*, 2006; Abelson *et al.*, 2007; Peterson *et al.*, 2007; Garza *et al.*, 2009; Weiss *et al.*, 2010; Restall *et al.*, 2011; Fischer *et al.*, 2013; Brandstetter *et al.*, 2014; Regier *et al.*, 2014; Truiett-Theodorson *et al.*, 2015; Damani *et al.*, 2016; Barbieri *et al.*, 2018; Scheele *et al.*, 2019) |
| *Global/mutli-country* | (Peersman *et al.*, 2009) |

Supplementary table 2 Included studies categorized by WHO region.

| WHO region | Study |
| --- | --- |
| *AFRO* | (O’Meara *et al.*, 2011; Owusu, 2011; Shayo *et al.*, 2012; Pancras, 2016; Kapiriri, 2017; Madon *et al.*, 2018; Atinga *et al.*, 2019; Owusu *et al.*, 2019; Masefield *et al.*, 2020; Paul *et al.*, 2020; Vian *et al.*, 2020, 2022) |
| *EMRO* | (Anwari *et al.*, 2015) |
| *EURO* | (von dem Knesebeck *et al.*, 2002; Andersson *et al.*, 2005; South *et al.*, 2005; Rosen, 2006; Simonsen-Rehn *et al.*, 2006; Fischer *et al.*, 2013; Barbieri *et al.*, 2018; Scheele *et al.*, 2019) |
| *PAHO* | (Wallerstein, 2000; Boyce, 2001; Mosquera *et al.*, 2001; Yassi *et al.*, 2003; Arredondo and Orozco, 2006, 2008; Abelson *et al.*, 2007; Peterson *et al.*, 2007; Garza *et al.*, 2009; Weiss *et al.*, 2010; Restall *et al.*, 2011; Ruano, 2013; Regier *et al.*, 2014; Arredondo *et al.*, 2015; Truiett-Theodorson *et al.*, 2015; Damani *et al.*, 2016; Buele *et al.*, 2020) |
| *SEARO* | (Bishai *et al.*, 2002; Gurung and Tuladhar, 2013; Soares, 2013; Rao *et al.*, 2017; Gurung *et al.*, 2018; Rajan *et al.*, 2019) |
| *WPRO* | (Ramiro *et al.*, 2001; Eyre and Gauld, 2003; Brandstetter *et al.*, 2014) |
| *Global* | (Peersman *et al.*, 2009) |

Supplementary table 3: Included study categorized by the level of government assessed by the research tools.

| Level of government for study focus | Study |
| --- | --- |
| *Communities* | (Wallerstein, 2000; von dem Knesebeck *et al.*, 2002; Eyre and Gauld, 2003; Peterson *et al.*, 2007; Gurung and Tuladhar, 2013; Atinga *et al.*, 2019; Buele *et al.*, 2020; Vian *et al.*, 2022) |
| *Municipalities* | (Ramiro *et al.*, 2001; Yassi *et al.*, 2003; South *et al.*, 2005; Simonsen-Rehn *et al.*, 2006; Ruano, 2013; Truiett-Theodorson *et al.*, 2015; Rao *et al.*, 2017; Barbieri *et al.*, 2018; Scheele *et al.*, 2019) |
| *District* | (Mosquera *et al.*, 2001; Bishai *et al.*, 2002; Andersson *et al.*, 2005; Garza *et al.*, 2009; O’Meara *et al.*, 2011; Owusu, 2011; Shayo *et al.*, 2012; Pancras, 2016; Kapiriri, 2017; Gurung *et al.*, 2018; Madon *et al.*, 2018) |
| *Provincial; District* | (Anwari *et al.*, 2015) |
| *Province/State* | (Boyce, 2001; Arredondo and Orozco, 2006, 2008; Rosen, 2006; Abelson *et al.*, 2007; Weiss *et al.*, 2010; Restall *et al.*, 2011; Brandstetter *et al.*, 2014; Regier *et al.*, 2014; Arredondo *et al.*, 2015; Damani *et al.*, 2016; Vian *et al.*, 2020) |
| *National, district* | (Soares, 2013; Owusu *et al.*, 2019) |
| *National* | (Peersman *et al.*, 2009; Fischer *et al.*, 2013; Rajan *et al.*, 2019; Masefield *et al.*, 2020; Paul *et al.*, 2020) |
