# Supplementary material for: Assessing the interactions of people and policy-makers in social participation for health: an inventory of participatory governance measures from a rapid systematic literature review
Source: Int J Equity Health. 2023 Nov 17;22:240. doi: 10.1186/s12939-023-01918-2 (PMC10657134; doi:10.1186/s12939-023-01918-2)
Supplement: Supplementary file 3 — Additional file 3. [file 12939_2023_1918_MOESM3_ESM.docx]

Supplementary materials table 2 Inventory of all measures

| Social Partici-pation theme | Sub theme | Concept | Measure | Measure type | Data type | Ref |
| --- | --- | --- | --- | --- | --- | --- |
| Capacities | Government communication skills | Ability to listen | Discourse analysis of how community representatives are listened to when being on a committee | Qualitative | Document review | Brandstetter et al. 2014 |
| Capacities | Government communication skills | Ability to listen | Perceived mutual respect understanding and trust | Quantitative | Survey | Truiett-Theodorson et al. 2015 |
| Capacities | Government communication skills | Ability to negotiate with civil society | How does a community perceive levels of conflict resolution skills? | Qualitative | Interview | Wallerstein et al. 2000 |
| Capacities | Government communication skills | Ability to negotiate with civil society | Ability to compromise | Quantitative | Survey | Truiett-Theodorson et al. 2015 |
| Capacities | Government communication skills | Ability to provide feedback | Transparency assessment of reporting | Qualitative | Document review | Vian et al. 2022 |
| Capacities | Government communication skills | Clarity of communication | Clarity of background material | Qualitative | Survey | Abelson et al. 2007 |
| Capacities | Government communication skills | Clarity of communication | Clarity of meeting purpose | Qualitative | Survey | Abelson et al. 2007 |
| Capacities | Government communication skills | Clarity of communication | Communication between Health Facility Operation and Management Committees and the wider community | Qualitative | Interview | Gurung et al. 2018 |
| Capacities | Government communication skills | Clarity of communication | Presentations provided me with the background knowledge and understanding to contribute to the meeting. My voice was heard when discussing the policy directions (Quantitative Survey Participant evaluation (statement with 1-5 point likert scale) | Quantitative | Survey | Damani et al. 2016 |
| Capacities | Government communication skills | Clarity of communication | Whether effective communication strategies are in place that allow flow of information between the organization and its communities | Quantitative | Survey | Yassi et al. 2003 |
| Capacities | Government communication skills | Public awareness of the participatory space | % of public aware of existing priority-setting process | Quantitative | Survey | Kapiriri et al. 2017 |
| Capacities | Government communication skills | Public awareness of the participatory space | Awareness of participation mechanism | Quantitative | Survey | Mosquera et al. 2001 |
| Capacities | Government communication skills | Public awareness of the participatory space | Did you know of the call to the participatory budgeting assemblies? Do you think that the calls to the participatory budgeting assemblies were made by the appropriate means? | Quantitative | Survey | Buele et al. 2020 |
| Capacities | Government communication skills | Public awareness of the participatory space | Knowledge about a person or group that represents user interest | Quantitative | Survey | Mosquera et al. 2001 |
| Capacities | Government communication skills | Public awareness of the participatory space | Knowledge of some form of collective participation | Quantitative | Survey | Mosquera et al. 2001 |
| Capacities | Government communication skills | Public awareness of the participatory space | Knowledge of the new participatory mechanisms | Quantitative | Survey | Mosquera et al. 2001 |
| Capacities | Government communication skills | Public awareness of the participatory space | Knowledge of what a user association is | Quantitative | Survey | Mosquera et al. 2001 |
| Capacities | Government recognition skills | Perceived added value of participation | Meaning attributed to citizen-user involvement | Qualitative | Interview | Restall et al. 2011 |
| Capacities | Government recognition skills | Perceived added value of participation | Perceived government views on participation | Qualitative | Interview | Boyce et al. 2001 |
| Capacities | Government recognition skills | Perceived added value of participation | The quality of representation is at the crux of the quality of the National Health Assembly. | Qualitative | Focus group discussion or in-depth interview | Rajan et al. 2019 |
| Capacities | Government recognition skills | Perceived added value of participation | The value of bringing together a wide range of stakeholders to discuss complex health challenges on a regular basis. It is recognised as a national public good. | Qualitative | Interview | Rajan et al. 2019 |
| Capacities | Government recognition skills | Perceived added value of participation | I believe in including those who are affected by Provincial Public Health Coordination Committee decision or their representatives in the Provincial Public Health Coordination Committee decision-making process (Scale of 1-10). | Quantitative | Survey | Anwari et al. 2015 |
| Capacities | Government recognition skills | Perceived added value of participation | I include those who are affected by Provincial Public Health Coordination Committee decisions or their representatives in the Provincial Public Health Coordination Committee decision-making process. (Scale of 1-10). | Quantitative | Survey | Anwari et al. 2015 |
| Capacities | Government recognition skills | Perceived added value of participation | Members see collaboration as in their self-interest | Quantitative | Survey | Truiett-Theodorson et al. 2015 |
| Capacities | Government technical skills | Participant perception of facilitation | How are patients views captured? | Qualitative | Interview | Owusu et al. 2019 |
| Capacities | Government technical skills | Participant perception of facilitation | How governance committee members answer questions posed by external stakeholders, looking at 3 criteria: demonstrated understanding of the question, a straightforward and clear answer, and justification or additional detail to support the answer | Qualitative | Document review | Vian et al. 2022 |
| Capacities | Government technical skills | Participant perception of facilitation | Number of opportunities each stakeholder gets to express opinion | Qualitative | Document review | Kapiriri et al. 2017 |
| Capacities | Government technical skills | Participant perception of facilitation | Organizing participation in health at the municipal level | Qualitative | Discussion | Ruano et al. 2013 |
| Capacities | Government technical skills | Participant perception of facilitation | What mechanisms were used, or should have been used, to enhance meaningful community involvement? | Qualitative | Interview | Yassi et al. 2003 |
| Capacities | Government technical skills | Participant perception of facilitation | I had the opportunity to share ideas and issues. | Quantitative | Survey | Damani et al. 2016 |
| Capacities | Government technical skills | Participant perception of facilitation | Inclusion of decision-making was defined as the percentage of Health Facility Operation and Management Committees meetings at which at least one dalit or woman member raised issues. | Quantitative | Survey | Gurung et al. 2013 |
| Capacities | Government technical skills | Participant perception of facilitation | Partnership Involvement Facilitation; This scale was a composite index, composed of five key leadership and management items, and it assessed the degree to which partnership leadership was effective in (a) working to develop a common language within the partnership; (b) fostering respect, trust, inclusiveness, and openness in the partnership; (c) creating an environment here differences of opinion can be voiced; (d) minimizing the barriers to participation in the partnership’s meetings and activities; and (e) preparing materials that inform members and help them make timely decisions. All items were scored on a 5-point scale (1 to 5), with 5 corresponding to excellent and 1 corresponding to poor. The score for the Partnership Involvement Facilitation scale was the average of these five items; the possible scale score range was 1.0 to 5.0 | Quantitative | Survey | Weiss et al. 2010 |
| Capacities | Government technical skills | Participant perception of space design | Barriers to participation | Qualitative | in-depth interview | Mosquera et al. 2001 |
| Capacities | Government technical skills | Participant perception of space design | By looking at the local conditions here, had it not been the barriers you have just told me, what existing factors do you think potentially could have contributed in making it easier for you as the local health authority to promote community participation in this district? Will you explain the significance of these factors? | Qualitative | Interview | Owusu et al. 2011 |
| Capacities | Government technical skills | Participant perception of space design | From your experience, what are the common problems faced by Health Facility Governing Committees in the course of accomplishing their roles? Mention the major four problems they faced. | Qualitative | Interview | Pancras et al. 2016 |
| Capacities | Government technical skills | Participant perception of space design | From your own perspectives, what have been the existing challenges/barriers that often prevent the communities from fully taking part in the malaria control decision making process? | Qualitative | Interview | Owusu et al. 2011 |
| Capacities | Government technical skills | Participant perception of space design | How are stakeholders invited? | Qualitative | Interview | Owusu et al. 2019 |
| Capacities | Government technical skills | Participant perception of space design | If you put these barriers or problems in this community aside, what local existing factors do you think could have made it easier for the local health authority to promote community participation in this district? | Qualitative | Interview | Owusu et al. 2011 |
| Capacities | Government technical skills | Participant perception of space design | Informants were asked about the level of women’s representation in and contribution to decision-making bodies, the extent to which their views were taken into consideration, how women’s participation was perceived and potential barriers to their participation and influence. | Qualitative | Interview | Shayo et al. 2012 |
| Capacities | Government technical skills | Participant perception of space design | Initiatives to promote greater participation | Qualitative | Interview | Arredondo et al. 2015 |
| Capacities | Government technical skills | Participant perception of space design | Mechanism to ensure balance of powers | Qualitative | Mixed-methods | Paul et al. 2020 |
| Capacities | Government technical skills | Participant perception of space design | Number of stakeholders: must be manageable for meaningful dialogue | Qualitative | Mixed-methods | Paul et al. 2020 |
| Capacities | Government technical skills | Participant perception of space design | Perceived participatory strategies | Qualitative | Interview | Boyce et al. 2001 |
| Capacities | Government technical skills | Participant perception of space design | The depth of participation in Health Facility Operation and Management Committees | Qualitative | Interview | Gurung et al. 2018 |
| Capacities | Government technical skills | Participant perception of space design | What have been the challenges/barriers that the local health authorities face in their attempt to promote community participation in this district? | Qualitative | Interview | Owusu et al. 2011 |
| Capacities | Government technical skills | Participant perception of space design | What the interviewee thought of the nature and extent of community participation | Qualitative | Interview | Yassi et al. 2003 |
| Capacities | Government technical skills | Participant perception of space design | Effective Health Facility Operation and Management Committees meeting (a prepared action plan) | Quantitative | Survey | Gurung et al. 2013 |
| Capacities | Government technical skills | Participant perception of space design | Initiatives to promote greater participation | Quantitative | Interviews | Arredondo et al. 2008 |
| Capacities | Government technical skills | Participant perception of space design | Were mechanisms implemented so that people with disabilities can attend the assemblies? | Quantitative | Survey | Buele et al. 2020 |
| Capacities | Population communication skills | Ability to negotiate | Ability to compromise | Quantitative | Survey | Truiett-Theodorson et al. 2015 |
| Capacities | Population communication skills | Ability to speak publicly | Discourse analysis of how community representatives communicate when being on a committee | Qualitative | Document review | Brandstetter et al. 2014 |
| Capacities | Population communication skills | Ability to speak publicly | Inclusion of decision-making was defined as the percentage of Health Facility Operation and Management Committees meetings at which at least one dalit or woman member raised issues. | Quantitative | Survey | Gurung et al. 2013 |
| Capacities | Population recognition skills | Perceived usefulness of participation | Benefits of participatory mechanisms | Qualitative | in-depth interview | Mosquera et al. 2001 |
| Capacities | Population recognition skills | Perceived usefulness of participation | Do you believe community participation is the key to the success of malaria control in this district? What are your reasons? | Qualitative | Interview | Owusu et al. 2011 |
| Capacities | Population recognition skills | Perceived usefulness of participation | Meaning attributed to citizen-user involvement | Qualitative | Interview | Restall et al. 2011 |
| Capacities | Population recognition skills | Perceived usefulness of participation | Perceived purpose of participation | Qualitative | Interview | Boyce et al. 2001 |
| Capacities | Population recognition skills | Perceived usefulness of participation | The quality of representation is at the crux of the quality of the National Health Assembly. | Qualitative | Focus group discussion or in-depth interview | Rajan et al. 2019 |
| Capacities | Population recognition skills | Perceived usefulness of participation | Member satisfaction with role, “How satisfied are you with your role in the partnership?” | Quantitative | Survey | Weiss et al. 2010 |
| Capacities | Population recognition skills | Perceived usefulness of participation | Members see collaboration as in their self-interest | Quantitative | Survey | Truiett-Theodorson et al. 2015 |
| Capacities | Population recognition skills | Perceived usefulness of participation | Perceived usefulness of the space to improve co-ordination of health and social care | Quantitative | Survey | Von Dem Knesebeck et al. 2002 |
| Capacities | Population recognition skills | Perceived usefulness of participation | Should the general public be offered more opportunities to influence health care resource allocation? | Quantitative | Survey | Rosen et al. 2006 |
| Capacities | Population recognition skills | Perceived usefulness of participation | Should the general public participate in discussions on health care resource allocation? | Quantitative | Survey | Rosen et al. 2006 |
| Capacities | Population recognition skills | Perceived usefulness of participation | The meeting was valuable to attend, the meeting was relevant to me or my work | Quantitative | Survey | Damani et al. 2016 |
| Capacities | Population recognition skills | Perception of empowerment | Do you think there is an opportunity for you at your level to get involved in health policy-making? | Qualitative | in-depth interviews | Masefield et al. 2020 |
| Capacities | Population recognition skills | Perception of empowerment | Self evaluation of "being listened to, self esteem, confidence, advocacy skills | Qualitative | Interview | Brandstetter et al. 2014 |
| Capacities | Population recognition skills | Perception of empowerment | Sense of community and ownership of participatory space | Qualitative | Interview | Atinga et al. 2019 |
| Capacities | Population recognition skills | Perception of empowerment | Perceived feelings of empowerment | Quantitative | Survey | Ramiro et al. 2001 |
| Capacities | Population recognition skills | Perception of empowerment | Perceived mutual respect, understanding and trust | Quantitative | Survey | Truiett-Theodorson et al. 2015 |
| Capacities | Population technical skills | Capacity to engage | Adequate capacities | Qualitative | Mixed-methods | Paul et al. 2020 |
| Capacities | Population technical skills | Capacity to engage | From your experience, what are the common problems faced by Health Facility Governing Committees in the course of accomplishing their roles? Mention the major four problems they faced? | Qualitative | Interview | Pancras et al. 2016 |
| Capacities | Population technical skills | Capacity to engage | How do you build the capacity of Health Facility Governing Committees members in the District Council? | Qualitative | Interview | Pancras et al. 2016 |
| Capacities | Population technical skills | Capacity to engage | How does a community express the characteristics and principles of a healthier community (such factors as vision, levels of collaboration and participation, coalition diversity, conflict resolution skills, political leadership, healthy policies, and addressing inequities)? What are the barriers to and facilitators of change? | Qualitative | in-depth interview | Wallerstein et al. 2000 |
| Capacities | Population technical skills | Capacity to engage | Prior activity related to deliberation issues (thinking , reading, research, talking) | Qualitative | Survey | Abelson et al. 2007 |
| Capacities | Population technical skills | Capacity to engage | What is the capacity of stakeholders to advocate and be involved effectively with government in the development of policies, plans and budgets for health services? | Qualitative | Focus group discussion or in-depth interview | Soares et al. 2013 |
| Capacities | Population technical skills | Capacity to engage | What is your lever of participation and understanding on each of the roles? | Quantitative | Survey | Pancras et al. 2016 |
| Capacities | Population technical skills | Capacity to engage | Did you receive any training regarding your roles/responsibilities from District level? | Quantitative | Survey | Pancras et al. 2016 |
| Capacities | Population technical skills | Capacity to engage | Were you trained to participate in participatory budgeting assemblies? | Quantitative | Survey | Buele et al. 2020 |
| Capacities | Population technical skills | Technical knowledge of the issue | Participant understanding of deliberation issue | Qualitative | Survey | Abelson et al. 2007 |
| Capacities | Population technical skills | Technical knowledge of the issue | I understood the material presented | Quantitative | Survey | Damani et al. 2016 |
| Legal framework | N/A | Documented procedures and strategies for participation | Current policy/strategy of intersectoral collaboration in terms of the way it is used to promote community participation? | Qualitative | Interview | Owusu et al. 2011 |
| Legal framework | N/A | Documented procedures and strategies for participation | Documented strategy to enlist public preferences | Qualitative | Document review | Kapiriri et al. 2017 |
| Legal framework | N/A | Documented procedures and strategies for participation | Whether organizational procedures facilitate participation | Quantitative | Observation | South et al. 2005 |
| Legal framework | N/A | Participatory spaces delineated in laws and programs | Level of collaboration: a) Obligating partnership (i.e. there exists a formal mutually committing agreement) b) Co-ordinated collaboration (i.e. there exists agreed-upon principles for the collaboration, which the actors can choose to follow) c) The municipality asks for our opinion (e.g. in connection with conventional hearing procedures) d) The municipality informs us (i.e. one-way communication from the municipality to the association) e) The municipality does not involve us | Qualitative | Survey | Scheele et al. 2018 |
| Legal framework | N/A | Participatory spaces delineated in laws and programs | Spaces for community participation in governmental programs | Qualitative | Interview | Arredondo et al. 2015 |
| Legal framework | N/A | Participatory spaces delineated in laws and programs | Existence of stable participatory structures for the implementation of community-based interventions (e.g., community development plans, etc.) | Quantitative | Interview + Document review | Barbieri et al. 2018 |
| Legal framework | N/A | Participatory spaces delineated in laws and programs | Identification of legal and normative frameworks | Quantitative | Interviews | Arredondo et al. 2008 |
| Policy uptake | N/A | Documented impact on decision-making | Percentage of programmes endorsed by participatory space being implemented | Quantitative | Document review | Von Dem Knesebeck et al. 2002 |
| Policy uptake | N/A | Documented impact on decision-making | Proportion of decisions reflecting public values | Quantitative | Document review | Kapiriri et al. 2017 |
| Policy uptake | N/A | Documented impact on decision-making | Sub item on type of evidence for decision making: expert opinion, patient input, input from general public or lay public | Quantitative | Survey | Regier et al. 2014 |
| Policy uptake | N/A | Link to downstream changes | Retracing a chronological link between the community participation and change | Qualitative | Document review | Brandstetter et al. 2014 |
| Policy uptake | N/A | Link to downstream changes | Percentage of increase in gram panchayat (village council) activities for health | Quantitative | Randomized Controlled Trial | Rao et al. 2017 |
| Policy uptake | N/A | Link to downstream changes | Percentage of target increase for indicator prioritised by committees vs. Percentage of target increase for other indicators | Quantitative | Document review | O'Meara et al. 2011 |
| Policy uptake | N/A | Link to downstream changes | Percentage of communities with budget allocation as a result of Participatory Budgeting | Quantitative | Document review | Buele et al. 2020 |
| Policy uptake | N/A | Perceived impact on decision-making | Are you aware of the way decisions on malaria control are made in this district? Can you share with me how malaria control policymaking process usually takes place? For example, how does the planning take place, who implement them and how is it monitored and evaluated? | Qualitative | Interview | Owusu et al. 2011 |
| Policy uptake | N/A | Perceived impact on decision-making | Capacity of participatory mechanisms to lead to change | Qualitative | in-depth interview | Mosquera et al. 2001 |
| Policy uptake | N/A | Perceived impact on decision-making | Do you collaborate with the government by influencing health policy and/or budgeting?  A. If yes, to what extent were you and your organisation involved in the development of the national health policy and health sector strategic plan? B. If yes, to what extent were you and your organisation involved in the development of the health budget? | Qualitative | in-depth interviews | Masefield et al. 2020 |
| Policy uptake | N/A | Perceived impact on decision-making | How was it decided that this community would use/do (insert name of project)? b. Were you involved in that decision? c. If yes, tell me how. d. If no, why not? Who was involved and how? | Qualitative | Case study | Peterson et al. 2007 |
| Policy uptake | N/A | Perceived impact on decision-making | In your own view, explain to me the way the community members are allowed to take part in the decision-making process? Will you say that the community members are fully allowed by the health authorities to participate in the process? What are your reasons for saying that? | Qualitative | Interview | Owusu et al. 2011 |
| Policy uptake | N/A | Perceived impact on decision-making | Needs assessment impact 1-5 scale a) Services dictated from external health agency. Medical/ professional view predominates. b) Medical interests determine all services, with minimal consideration of community views. c) Active representation of community views and assessment of community health service needs in service decisions. d) The community health organization uses community needs assessment information e) Community members in general are involved in broad health needs assessment, and in decision-making and implementation of solutions. | Qualitative | Interview | Eyre et al. 2003 |
| Policy uptake | N/A | Perceived impact on decision-making | participation in program design and decision making | Qualitative | Interview | Arredondo et al. 2015 |
| Policy uptake | N/A | Perceived impact on decision-making | Perceived local participation in decision making | Qualitative | Interview | Arredondo et al. 2006 |
| Policy uptake | N/A | Perceived impact on decision-making | Perceived participation of social actors of the municipal level in the allocation of resources according to local needs | Qualitative | Interview | Arredondo et al. 2006 |
| Policy uptake | N/A | Perceived impact on decision-making | Perception of local participation in decision making | Qualitative | Interview | Arredondo et al. 2006 |
| Policy uptake | N/A | Perceived impact on decision-making | Perception of participation of social actors of the municipal level in the allocation of resources according to local needs | Qualitative | Interview | Arredondo et al. 2006 |
| Policy uptake | N/A | Perceived impact on decision-making | The decision-making process | Qualitative | In-depth intervivews and focus group discussions | Garza et al. 2009 |
| Policy uptake | N/A | Perceived impact on decision-making | To what extent are the private sector, civil society and stakeholders consulted in health services decision making? | Qualitative | Focus group discussion or in-depth interview | Soares et al. 2013 |
| Policy uptake | N/A | Perceived impact on decision-making | What major role (s) do you perceive the community members to be playing in malaria control programme activities? | Qualitative | Interview | Owusu et al. 2011 |
| Policy uptake | N/A | Perceived impact on decision-making | When it comes to malaria control policymaking process, in what way(s) are the communities allowed to take part in all the stages of the process? In your opinion, is it fair to say that the community members have often been allowed to participate fully (i.e. in all the stages) in the malaria control decision making process? Why? | Qualitative | Interview | Owusu et al. 2011 |
| Policy uptake | N/A | Perceived impact on decision-making | Who has influence on legislation pertaining to health? | Qualitative | Focus group discussion or in-depth interview | Soares et al. 2013 |
| Policy uptake | N/A | Perceived impact on decision-making | Community involvement in decision-making | Quantitative | Interviews | Arredondo et al. 2008 |
| Policy uptake | N/A | Perceived impact on decision-making | Extent of civil society involvement in national planning and budgeting processes (scale 1-5) and the review of the national acquired immunodeficiency syndrome strategy (scale 1-5) | Quantitative | Survey | Peersman et al. 2009 |
| Policy uptake | N/A | Perceived impact on decision-making | Member satisfaction with influence; “How satisfied are you with your influence in the partnership?” | Quantitative | Survey | Weiss et al. 2010 |
| Policy uptake | N/A | Perceived impact on decision-making | National Acquired Immunodeficiency Syndrome Strategy was developed with active participation of civil society | Quantitative | Survey | Peersman et al. 2009 |
| Policy uptake | N/A | Perceived impact on decision-making | Our association has influence on decision-making concerning health matters in the municipality | Quantitative | Survey | Simonsen-Rehn et al. 2006 |
| Policy uptake | N/A | Perceived impact on decision-making | Participation in programme design and in decision making | Quantitative | Interviews | Arredondo et al. 2008 |
| Policy uptake | N/A | Perceived impact on decision-making | Perceived ability to influence decisions within the system | Quantitative | Survey | Mosquera et al. 2001 |
| Policy uptake | N/A | Perceived impact on decision-making | Perceived level of involvement and level of influence in health decisions. | Quantitative | Survey | Ramiro et al. 2001 |
| Policy uptake | N/A | Perceived impact on decision-making | Whether communities are involved in the range of decision-making taking place in the organization | Quantitative | Observation | South et al. 2005 |
| Representativeness | N/A | Diversity | Numbers and types of participants | Qualitative | Interview | Boyce et al. 2001 |
| Representativeness | N/A | Diversity | Participant characteristics: Age, sex, education; Length of residency; Participation in community organizations; Experience with the issue under deliberation | Qualitative | Interview | Abelson et al. 2007 |
| Representativeness | N/A | Diversity | Relevant stakeholders, including health service providers | Qualitative | Mixed-methods | Paul et al. 2020 |
| Representativeness | N/A | Diversity | Relevant stakeholders, including policymakers and health planners (yes/no) | Qualitative | Mixed-methods | Paul et al. 2020 |
| Representativeness | N/A | Diversity | Relevant stakeholders, including the various levels of the health system | Qualitative | Mixed-methods | Paul et al. 2020 |
| Representativeness | N/A | Diversity | Which interest groups are represented in the local committee? How were the committee members recruited? Who decides the course of action? | Qualitative | Interview | Andersson et al. 2005 |
| Representativeness | N/A | Diversity | AIDS coordinating body includes civil society representative(s) (Yes/No) | Quantitative | Survey | Peersman et al. 2009 |
| Representativeness | N/A | Diversity | Composition of the Social Services Committee in the intervention wards before and during study implementation. | Quantitative | Document review | Madon et al. 2018 |
| Representativeness | N/A | Diversity | Effective HFOMC meeting (The meeting needs to have a participation of 51% of Health Facility Operation and Management Committees members with at least a dalit and woman member) | Quantitative | Document review and observation | Gurung et al. 2013 |
| Representativeness | N/A | Diversity | How many female members are in your Health Facility Governing Committees? | Quantitative | Survey | Pancras et al. 2016 |
| Representativeness | N/A | Diversity | Number of stakeholders participating and number and type of members from the general public represented | Quantitative | Document review | Kapiriri et al. 2017 |
| Representativeness | N/A | Diversity | Organisations represented in the local steering committees | Quantitative | Document review | Andersson et al. 2005 |
| Representativeness | N/A | Diversity | sub items related to different type of publics: general or lay public patients professionals industry/or lobbyists patient representatives/advocates | Quantitative | Survey | Regier et al. 2014 |
| Representativeness | N/A | Diversity | Traditional Leadership grouping includes chief representatives, senior headmen, headmen, and section chairmen; Health Volunteers includes members of the Neighborhood Health Committee, Safe Motherhood Action Group, and community health workers; Government includes civic counselor at ward level; Other community members includes farmers and those who did not state occupation or affiliation. | Quantitative | observation and analysis of written documentation | Vian et al. 2020 |
| Representativeness | N/A | Diversity | Whether community diversity is reflected in the organization and its processes | Quantitative | Observation | South et al. 2005 |
| Representativeness | N/A | Participant perception of clarity of roles | Are you involved in malaria control activities? What major role (s) do you perceive yourself or any other community members play in malaria control programme activities? | Qualitative | Interview | Owusu et al. 2011 |
| Representativeness | N/A | Participant perception of clarity of roles | interviewee’s perception of the role of the various organizations | Qualitative | Interview | Yassi et al. 2003 |
| Representativeness | N/A | Participant perception of clarity of roles | Who are those responsible for activities being implemented? What role has the local steering committee? | Qualitative | Interview | Andersson et al. 2005 |
| Representativeness | N/A | Participant perception of clarity of roles | Effective Health Facility Operation and Management Committeesmeeting (shared responsibilities among members) | Quantitative | Survey | Gurung et al. 2013 |
| Representativeness | N/A | Participant perception of the quality of representation | how citizen-users have been represented in policy development | Qualitative | Interview | Restall et al. 2011 |
| Representativeness | N/A | Participant perception of the quality of representation | How does a community perceive levels of diversity and addressing inequities? | Qualitative | Interview | Wallerstein et al. 2000 |
| Representativeness | N/A | Participant perception of the quality of representation | Leadership 1-5 scale a) One sided (i.e. autocratic chairperson on community organization driving decisions or health staff assume leadership), or vested interests in leadership undemocratically chosen and unrelated to community interests. b) Community organization representative of community, but health staff work independently of organization. c) Community organization functioning in collaboration with health staff, but without wide support from the community. d) Active community organization but acknowledges lack of input/representation from marginalized section of the community. Tendency towards lack of heterogeneity amongst the leadership. e) Community organization fully represents variety of interests in community and is motivated out of concern for the health of all its population in the future. | Qualitative | Interview | Eyre et al. 2003 |
| Representativeness | N/A | Participant perception of the quality of representation | Perception of participation of non-governmental organization leaders | Qualitative | Interview | Arredondo et al. 2006 |
| Representativeness | N/A | Participant perception of the quality of representation | Perception of representation | Qualitative | in-depth interview | Mosquera et al. 2001 |
| Representativeness | N/A | Participant perception of the quality of representation | Perception of the role of community leaders in health matters | Qualitative | Interview | Arredondo et al. 2006 |
| Representativeness | N/A | Participant perception of the quality of representation | Relevant stakeholders, including clients/citizens // those individuals affected // engagement on the part of (staff and) the public // population / beneficiaries // community representatives | Qualitative | Mixed-methods | Paul et al. 2020 |
| Representativeness | N/A | Participant perception of the quality of representation | The selection of members | Qualitative | Interview | Gurung et al. 2018 |
| Representativeness | N/A | Participant perception of the quality of representation | What the interviewee thought of the participation of various groups, particularly the role of women | Qualitative | Interview | Yassi et al. 2003 |
| Representativeness | N/A | Participant perception of the quality of representation | Will you say that other governmental sectors or private actors have been actively involved in your district? What makes you say that and can you explain to me? | Qualitative | Interview | Owusu et al. 2011 |
| Representativeness | N/A | Participant perception of the quality of representation | Collaboration group seen as a legitimate leader in the community | Quantitative | Survey | Truiett-Theodorson et al. 2015 |
| Representativeness | N/A | Participant perception of the quality of representation | Cooperation with municipal spheres of authority | Quantitative | Survey | Simonsen-Rehn et al. 2006 |
| Representativeness | N/A | Participant perception of the quality of representation | Perceived extent of participation in health activities | Quantitative | Survey | Ramiro et al. 2001 |
| Representativeness | N/A | Participant perception of the quality of representation | The meeting brought together relevant stakeholders | Quantitative | Survey | Damani et al. 2016 |
| Representativeness | N/A | Proportion of different stakeholder groups | Average proportion of VDC members who are Brahmi or highest caste in each facility | Quantitative | Document review | Bishai et al., 2002 |
| Representativeness | N/A | Proportion of different stakeholder groups | Average proportion of village development committee members who are Chatriya or second highest caste in each facility | Quantitative | Document review | Bishai et al., 2002 |
| Representativeness | N/A | Proportion of different stakeholder groups | Average proportion of village development committee members who are neither highest or second highest casste in each facility | Quantitative | Document review | Bishai et al., 2002 |
| Representativeness | N/A | Proportion of different stakeholder groups | Average proption of village development committee members who are women | Quantitative | Document review | Bishai et al. 2002 |
| Representativeness | N/A | Proportion of different stakeholder groups | Number of types of stakeholders involved in decision process. High diversity of stakeholders increases the possibility that particular interests of single stakeholders are balanced out. Types of stakeholders, equally weighed: Service provider(s) Payer Government Patients/patient representative(s) Industry | Quantitative | Survey | Fischer et al. 2013 |
| Representativeness | N/A | Proportion of different stakeholder groups | Proportion of lower caste represented in committee | Quantitative | Document review | Bishai et al. 2002 |
| Representativeness | N/A | Proportion of different stakeholder groups | We identified 7 possible external stakeholder groups (traditional leadership, health facility staff, health volunteers, church representatives, school staff, government officials, and other regular community members; described in Supplementary file 1) who had been invited to attend the annual general meeting. We calculated the percent represented at the meeting by at least 1 attendee per group. If an individual represented 2 groups, the chairperson asked that he or she only state one group. Calculations of participation proportions, medians, and ranges for stakeholder groups in attendance were conducted in Microsoft Excel | Quantitative | Document review | Vian et al. 2022 |
| Sustainability | N/A | History of participation | Continuity in community participation | Qualitative | Discussion | Ruano et al. 2013 |
| Sustainability | N/A | History of participation | Effective Health Facility Operation and Management Committeesmeeting (the percentage of Health Facility Operation and Management Committeesthat had held meetings in the previous month) | Quantitative | Survey | Gurung et al. 2013 |
| Sustainability | N/A | History of participation | History of collaboration or cooperation in the community | Quantitative | Survey | Truiett-Theodorson et al. 2015 |
| Sustainability | N/A | Political will | Local leadership support for participatory space | Qualitative | Interview | Atinga et al. 2019 |
| Sustainability | N/A | Resources for participation | Capacity building (incl. at subnational level) / Adequate technical support | Qualitative | Mixed-methods | Paul et al. 2020 |
| Sustainability | N/A | Resources for participation | How do you build the capacity of Health Facility Governing Committees members in the District Council? | Qualitative | Interview | Pancras et al. 2016 |
| Sustainability | N/A | Resources for participation | Screening budget for investment in participation and empowerment | Qualitative | Document review | Brandstetter et al. 2014 |
| Sustainability | N/A | Resources for participation | Did you receive any training regarding your roles/responsibilities from District level? | Quantitative | Survey | Pancras et al. 2016 |
| Sustainability | N/A | Resources for participation | Sufficient funds, materials and time | Quantitative | Survey | Truiett-Theodorson et al. 2015 |
| Sustainability | N/A | Resources for participation | What the organization does to support and develop staff to engage with communities | Quantitative | Observation | South et al. 2005 |
| Sustainability | N/A | Sustained attendance | Percentage of people that attended Gram/Ward Sabha (municipal open to all meeting, 2 to 4 times a year) | Quantitative | Survey | Rao et al. 2017 |
| Sustainability | N/A | Sustained attendance | Attendance of member (Mayor, municipal health officer, Department of Health representative, local councillor for health, non-governmantal organization representative) | Quantitative | Document review | Ramiro et al. 2001 |
| Sustainability | N/A | Sustained attendance | Average number of village development committtee members who attend regularly | Quantitative | Document review | Bishai et al. 2002 |
| Sustainability | N/A | Sustained attendance | Rate of participation in round tables for different stakeholder groups: Local government, Physicians associations, Health insurance funds, Hospitals, Charitable organisations, Political parties, Self help groups | Quantitative | Document review | Von Dem Knesebeck et al. 2002 |
